# Daphnetin Ameliorates the Amyloid β-Induced Alzheimer Disease via Restoring Potassium-Chloride Co-Transporter 2 (KCC2) Ion Channel Functions in Mice

**DOI:** 10.5812/ijpr-164601

**Published:** 2025-12-17

**Authors:** Yanyan Zhou, Fang Zhou, Hongbin Li

**Affiliations:** 1 Department of Neurology, Affiliated Hospital of Zunyi Mdical University, Zunyi, Guizhou, China; 2 Zunyi Medical University, Zunyi, Guizhou, China; 3 Department of General Medical, The People's Hospital of Dazu, Chongqing, China; 4 Second Department of Neurology, Tianjin Beichen Traditional Chinese Medicine Hospital, Tianjin, China

**Keywords:** Acetylcholinesterase, Morris Water Maze, Neurodegeneration, Novel Object Recognition Test, Y-Maze Alternation Task

## Abstract

**Background:**

Alzheimer’s disease (AD) is a chronic neurodegenerative disorder characterized by downregulation of potassium voltage-gated channel subfamily a member 2 (KCNA2) proteins. Potassium voltage-gated channel subfamily a member 2 is involved in the regulation of neuronal excitability by restoring neuronal potassium-chloride co-transporter 2 (KCC2) functions. Coumarin derivatives exert neuroprotective effects via upregulation of KCC2 proteins. Daphnetin (DPN; 7,8-dihydroxy coumarin) is a polyphenolic compound known to attenuate cognitive dysfunction. However, the role of DPN in the attenuation of AD-associated cognitive dysfunctions through regulation of KCC2 functions has not yet been investigated.

**Objectives:**

The present study was designed to investigate the role of DPN against amyloid-β oligomer-induced AD in mice.

**Methods:**

In this study, a total of six groups with eight male Swiss albino mice per group were used. The simple randomization method was adopted for unbiased assignment of animals based on age, sex, and weight variations. Alzheimer’s disease in mice was induced by intracerebroventricular (i.c.v.) injection of amyloid-β oligomer (Aβ; 4 μg/4 μL). The test compounds, i.e., DPN (40, 80, and 120 mg/kg of body weight), and donepezil (DP, 2 mg/kg), were administered orally (p.o.) for 21 consecutive days. Behavioral changes, including the Morris water maze (MWM) test, water Y-maze alternation test (WYMA), and novel object recognition test (NORT), were assessed according to the experimental protocol. Furthermore, hippocampal brain tissue biomarkers, namely acetylcholinesterase (AChE) activity, thiobarbituric acid reactive substances (TBARS), reduced glutathione (GSH), and KCC2 levels, were also estimated. In addition, Aβ-associated brain histopathological changes were evaluated using the eosin and hematoxylin staining method. Six mouse hippocampus tissue samples were used for the assessment of tissue biomarkers, and the remaining two brain tissues were used for histological observations. Behavioral data were statistically analyzed by two-way analysis of variance (ANOVA), and biomarkers were analyzed by one-way ANOVA. The 95% confidence level (P < 0.05) was set for confirmation of statistical significance.

**Results:**

The results revealed that administration of Aβ enhanced escape latency time (ELT) and reduced time spent in the target quadrant (TSTQ) values in the MWM test; increased transfer latency (TL) values in the WYMA test; and reduced percentage location preference (%LP) while increasing percentage Recognition Index (%RI) in the NORT test. Furthermore, Aβ induced increases in AChE activity and TBARS levels, along with reductions in GSH and KCC2 levels. It also caused neurodegeneration in the CA3 hippocampus region. However, DPN ameliorated the above Aβ-induced changes in cognitive behaviors, biomarkers, and histopathological levels.

**Conclusions:**

Daphnetin attenuates Aβ-associated AD progression via inhibition of AChE activity, scavenging of free radicals, reduction of inflammation, and restoration of neuronal KCC2 channels. Hence, it may be a potential therapeutic agent for the treatment of AD. However, more extensive studies are required to confirm this therapeutic potency in different AD conditions and various animal species.

## 1. Background

Alzheimer’s disease (AD) is a chronic organic brain disorder resulting from neurodegeneration, accumulation of amyloid and tau proteins, and formation of neurofibrillary tangles (1). The global prevalence rate of AD in 2021 exceeded 60 - 70% of the 57 million people in the dementia population. Furthermore, this rate increases every year with age 65 and doubles every five years (2). The projected rate of AD-type dementia is expected to reach 150 million by 2050 (3). The total lifetime cost of AD care was assessed at $250,174, representing a significant economic burden (4). The risk of AD progression is reduced by modifiable factors such as lifestyle, intake of micronutrients, and trace minerals, which are useful for the management of AD (5, 6). Major clinical risk factors, including chronic diabetic complications, cerebrovascular accidents, stroke, and cardiac failure, also contribute to the progression of AD (7).

The primary challenges for AD patients are serious memory loss, particularly loss of remembrance of recent events; difficulty performing familiar and everyday tasks; and decline in physical abilities (3). The molecular factors of AD pathogenesis include the abundant formation of beta-amyloid (Aβ) fragments and tau (τ) proteins, which lead to enhanced synaptic dysfunction, neurodegeneration, and cognitive decline (8, 9). In addition to beta-amyloid and tau proteins, certain growth factors such as epidermal growth factor and fibroblast growth factors, beta-site amyloid precursor protein cleaving enzyme-1, neprilysin, insulin-degrading enzymes, and ubiquitin conjugating enzyme E2B contribute to the chronic progression of AD (10). The aberrant activity of neuronal ion channels is a primary key player in the early progression of AD (11).

Potassium channels, especially Kv1.2 and Kv1.3, are highly expressed in microglia cells of the human brain with AD (12). The potassium voltage-gated channel subfamily a member 2 (KCNA2) gene variant is responsible for the expression of Kv1.2 channels in the nervous system, which regulate neuronal excitability (13). Potassium voltage-gated channel subfamily a member 2 genes encode the expression of neuronal potassium-chloride co-transporter 2 (KCC2), which plays a vital role in maintaining proper chloride ion extrusion within neurons (14). Moreover, modulation of KCNA2 can disrupt neuronal plasticity and synaptic functions, leading to intellectual disability, cognitive impairment, and epilepsy (15). In AD conditions, KCNA2 proteins are downregulated, leading to neuronal excitability and loss of the restorative function of the neuronal KCC2 ion channel (16). The downregulated function of KCC2 enhances the age-dependent rise of amyloid-β42 (Aβ42) peptide, with impaired chloride homeostasis and synaptic meta-plasticity (17). Experimentally, KCC2 enhancers such as CLP290 are known to restore KCC2 expression in various pathological conditions (18), hippocampal gamma-aminobutyric acid (GABA) neurotransmitter inhibition (19), and refractory neonatal seizures and epilepsy (20).

Natural compounds are known to produce neuroprotection in various experimental neurodegenerative models (21, 22). Coumarin derivatives exert neuroprotection via regulation of multiple ion channels and reduction of neuronal tau protein and Aβ40-42 peptide expression (23, 24). There is no literature report on the role of coumarin in the regulation of KCNA2 channels and KCC2 transporters. However, 7-hydroxycoumarin activates potassium channels, especially ATP-sensitive potassium channels, large-conductance calcium-activated potassium channels, and voltage-gated potassium channels (25). Furthermore, the coumarin derivative coumarsabin also regulates neuronal voltage-gated potassium channels, causing a reduction in neuronal excitability (26).

Furthermore, conventional AD drugs such as donepezil possess neuroprotective action via regulation of neuronal KCNQ2 and KCNQ3 channels (27) Restoration of neuronal KCC2 channel activity reverses cognitive decline in AD patients (16). The KCC2 transporter is widely expressed in various regions of the central nervous system, including the hippocampus. During brain development, abundant KCC2 expression occurs in the hippocampus (14). Furthermore, KCC2 is also found in the CA3 region of the hippocampus, which plays a crucial role in maintaining intracellular chloride homeostasis by enabling inhibitory GABAergic signaling, thereby reducing neuronal excitability and enhancing memory consolidation (28). Hence, the KCC2 ion channel is a novel drug target for the management of AD.

Daphnetin (DPN; 7,8-dihydroxy coumarin) is a polyphenolic coumarin derivative with potassium channel modulatory and broad neuroprotective actions (29). It also alleviates diabetes-associated cognitive dysfunction by maintaining blood-brain barrier integrity and modulation of glucagon-like peptide-1 receptor (30, 31). 

## 2. Objective

The role of DPN in the attenuation of Aβ-induced cognitive dysfunctions in relation to KCC2 functions has not yet been investigated. Hence, the present study was designed to investigate the role of DPN against Aβ oligomer-induced AD in mice.

## 3. Methods

### 3.1. Animals Used

Disease-free twelve-month-old male Swiss albino mice (20 to 30 grams) were used in this study. The animals had free access to food and water ad libitum. Animals were housed in the central animal house under standard laboratory conditions, i.e., twelve hours of daylight and darkness, 70 ± 1% relative humidity, and 23 ± 01°C (73 ± 01°F) temperature. This study design was approved by the Institutional Animal Ethics Committee (IAEC approval number: Z20250136; Dated: 01.12.2024). Animal care was conducted as per the University's IAEC guidelines.

### 3.2. Induction of Alzheimer Disease in Mice

Alzheimer’s disease in mice was induced by intracerebroventricular (i.c.v.) injection of Aβ1-42 oligomers (Aβ; 4 μg/4 μL) under anesthetic conditions as described by Paxinos and Franklin (32). Briefly, a combination of xylazine (5 mg/kg) and ketamine (75 mg/kg) was used to anesthetize the mice. The stereotactic device (Kent Scientific Corporation, Torrington, United States) was used to hold the animal. The brain epidermal layer was opened and sterilized using a 1% v/v povidone-iodine solution. The brain bregma and lambda regions of the skull were exposed. The lateral ventricle coordinates were marked using a stereotaxic device, and the skull was drilled with a bone micro-drilling device (Kent Scientific Corporation, Torrington, United States). The Hamilton syringe was applied to the lateral ventricle using stereotaxic coordinates: Anteroposterior: 0.2 mm; mediolateral: 1.0 mm; dorsoventral: 2.5 mm, as described by Park et al. (33). Two divided doses, i.e., 2 μL of 2 μg of Aβ oligomers, were injected into both lateral ventricles. Aβ oligomer solution was freshly prepared by dissolving the Aβ peptide in phosphate-buffered saline (1 μg/μL; pH = 7.5), and this preparation was incubated at 37°C for 3 days as described by Li et al. (34). The naive group received a 0.9% sodium chloride (normal saline) injection only. After administration, skull openings were closed with resin and allowed to harden, and the skin layer was sutured with monofilament (No.: 5-0) non-absorbable polypropylene suture (Ethicon Inc., New Jersey, United States). The topical antibiotic ointment (Fucidine 20 mg/g) was applied once. The animal was then kept for an additional hour in an individual ventilated cage to ensure complete recovery. Surgery was performed under controlled body temperature conditions (35ºC) using a rodent heating pad. Non-pharmacological intervention approaches were implemented to minimize pain, distress, and suffering in the animals. These non-pharmacological interventions included: (1) Acclimatization and habituation of the animals to the room, instruments, and handlers to reduce fear and stress; (2) provision of good husbandry and enrichment by ensuring proper housing, nutritional support, and social interaction with conspecifics to decrease anxiety; (3) skilled and refined handling of the animals to minimize tissue trauma and stress; and (4) slow injection to avoid excessive fluid leakage and the risk of shock.

Furthermore, this animal model fulfills the eligibility criteria for the selection of animal models that closely mimic human pathological conditions, including alterations in genetic patterns, biological processes, and disease progression. In addition, this model offers feasibility, low cost, reproducible data, and ethical acceptability for studies designed to address specific research questions (35).

### 3.3. Experimental Design

The experimental design consisted of six groups, each comprising eight mice. A simple randomization method was adopted to ensure unbiased assignment of animals to all experimental groups, taking into account baseline characteristics such as age (12 months old), sex (male), and weight (20 to 30 grams). This approach was used to ensure the unbiased allocation of animal responses without influencing the study's outcome. A Microsoft Excel-generated list was utilized to conduct the randomization process, which helped reduce bias, balance the groups, and validate the statistical analysis. The sample size was determined based on scientific rationale from literature reports and pilot study findings.

Animals in Group I served as naïve controls. Animals in Group II served as the negative control. Amyloid-β1–42 oligomer (Aβ; 4 μg/4 μL) was injected intracerebroventricularly (i.c.v.) into this group of mice to induce Alzheimer’s disease (AD). In AD-induced mice, animals in Groups III to V received oral (p.o.) treatment with DPN at doses of 40, 80, and 120 mg/kg, respectively, for 21 consecutive days. Group VI received donepezil (DP, 2 mg/kg) orally for 21 days.

The Morris water maze (MWM) test was used to assess cognitive behavioral alterations associated with AD from days 17 to 21. The water Y-maze alternation (WYMA) test was conducted from days 20 to 21, and the novel object recognition test (NORT) was performed from days 18 to 21. Behavioral alterations were recorded using a USB camera (12 Megapixel, Intex products, India), and the data were analyzed from pre-recorded video by manual observation.

On day 22, animals were sacrificed by cervical dislocation. The hippocampus region of the mouse brain tissue was collected for molecular studies. Hippocampal tissue biomarkers, including acetylcholinesterase (AChE; class of hydrolase; EC 3.1.1.7) activity, thiobarbituric acid reactive substances (TBARS), glutathione (GSH), and KCC2 levels, were estimated. Additionally, Aβ-associated brain histopathological changes were evaluated using the eosin and hematoxylin staining method. In each group, six animal tissue samples were used for biomarker assessment, while the remaining two brain tissues were used for histological observations.

A single-blinding procedure was implemented for data analysis, with the principal investigator being aware of the treatment group at each point of the experiment.

### 3.4. Assessment of Amyloid β1-42 Oligomer-Induced Spatial Cognitive Changes in Morris Water Maze Test

The MWM test was modified from the method described by Morris (36) with slight modification by Curdt et al. (37). The MWM test was performed on days 17 to 21. A hidden platform (10 × 10 cm; height 29 cm) was positioned in the Q4 quadrant of the circular MWM device (diameter: One hundred and fifty cm; height: Forty-five cm; water-filled to 30 cm). From days 17 to 20, animals were trained to recognize the platform location. Data from the 20th-day learning trial was used for quantification of escape latency time (ELT) between the normal group and data from the 17th day. The platform was identified in the Q4 quadrant on the 21st-day memory retention test, evaluated as time spent in the target quadrant (TSTQ). A non-toxic white dye was mixed into the water to make it opaque. The platform was removed, and animals were placed in the center of the apparatus. Ninety seconds was the cutoff time for ELT and TSTQ assessments. Animals failing to reach the platform during ELT evaluation were led to it and allowed to remain for ten seconds. All trials were conducted between 9:00 a.m. and 6:00 p.m., with water temperature at 37°C ± 1°C and illumination between 24 and 26 Lux. Table 1 details the learning and memory trial assessment plan.

**Table 1.** Planning of Learning and Memory Trial Assessments ^a^

Days	Order of Animal Placement in Quadrants				Assessments
**Day 17**	Q1	Q2	Q3	Q4	ELT
**Day 18**	Q2	Q3	Q4	Q1	
**Day 19**	Q3	Q4	Q1	Q2	
**Day 20**	Q4	Q1	Q2	Q3	
**Day 21**	The animal was placed at the center of the MWM device.				TSTQ

Abbreviations: ELT, escape latency time; Q, quadrant; TSTQ, time spent in the target quadrant.

^a^ The animal was allowed a ten-minute break between each quadrant trial during the ET trial test.

### 3.5. Assessment of Amyloid β1-42 Oligomer-Induced Cognitive Changes in Water Y-Maze Alternation Test

Cognitive function changes were assessed by the WYMA test as described by Deacon (38) with modification by Kraeuter et al. (39). The WYMA apparatus consisted of a 50 cm length, 40 cm width, and 20 cm height plastic chamber, with two short arms and one long arm (Y-shaped). The short arms were arranged at a 120º angle from the long arm. The right short arm was made with red plastic, the left with green plastic. The green chamber corner had a green square platform (10 × 10 cm, height 5 cm). Junctions (10 × 10 cm) were made with sky-blue plastic. The long arm was yellow plastic, with a 10 cm square compartment as the starting chamber. Water was maintained at 4 cm height, temperature at 24 ± 1°C. Mice were placed at the starting point and acclimatized for 5 minutes before testing. Guidance was provided if animals did not reach all arms. The next day, spatial cognitive function was assessed by determining transfer latency (TL), the time taken to reach the target platform. Higher TL indicates poorer cognitive function. The cutoff time was two minutes.

### 3.6. Assessment of Amyloid β1-42 Oligomer-Induced Cognitive Changes in Novel Object Recognition Test

Cognitive function changes were assessed by water NORT as described by Lueptow (40) with modification by Zhang et al. (41). The water NORT device was a solid plastic box (45 × 45 × 45 cm). The NORT assessment included prehabituation, habituation, training, and testing. Animals were acclimated for 30 minutes before the experiment on day 18, then allowed five minutes to explore the empty NORT box (pre-habituation). On days 19 and 20, animals habituated in the empty box for 20 minutes (habituation). On day 21, animals underwent a training trial with two identical objects placed in opposite corners, allowed to explore for 10 minutes. After one hour, a testing trial was performed with one familiar and one novel object, for 10 minutes. Objects were of similar size, different colors (red, yellow, green), and shapes (square, round, triangle), fixed to the box floor. These objects were securely fixed to the floor inside the NORT box to prevent displacement during both the training and testing phases. Additionally, after each animal exposure, the entire NORT box was thoroughly wiped with 70% v/v ethanol to eliminate any potential influence of olfactory cues on the NORT behavioral assessment. During the object exploration period, animal behaviors in the NORT were recorded as nose pointing (touching) the object, pawing, and sniffing the object. It should be noted that mere sniffing or vigorous vibrissae sweeping were not considered as object exploration activities. The NORT response was utilized to record the object exploration time.

The percentage location preference (%LP) was determined using the following formula, based on the time recorded during the training session for object exploration with two comparable objects:


$$\%LP\;=\frac{Time\;exploring\;one\;of\;the\;identical\;objects}{Time\;exploring\;the\;identical\;object\;pairs}\;\times 100$$

In the testing session, the object exploration time with one familiar object and one novel object was recorded as the percentage recognition index (%RI), calculated as follows:


$$\%RI\;=\frac{Time\;exploring\;the\;novel\;object}{Time\;exploring\;novel\;object\;+\;Time\;exploring\;familiar\;object}\;\times 100$$

A value of 50% LP in the normal control group was considered for this study, and any value of < 50% LP was interpreted as disregarding the influence of object location within the NORT box. If an animal exhibited a total exploration time of < 20 seconds during the testing phase, it was excluded from both %LP and %RI analyses in the NORT. Throughout the NORT response observation, laboratory environmental conditions were strictly maintained: Complete soundproofing, a temperature of 37 ± 1ºC, relative humidity in the range of 40 - 45%, and illumination of 24 - 26 Lux.

### 3.7. Estimations of Amyloid β1-42 Oligomer-Induced Tissue Markers Changes

Following the evaluation of behavioral measures, mice were sacrificed by cervical dislocation on day 22. The hippocampus region of the mouse brain was isolated according to the method described by Sultan (42). After isolation, the hippocampal brain tissue was homogenized in ice-cooled phosphate buffer (pH 7.4). The homogenate was then centrifuged at 769 G using an ultracentrifuge machine (Empire Bioscience Sdn Bhd, Selangor, Malaysia) to obtain a clear supernatant. Tissue indicators such as acetylcholinesterase (AChE) activity, thiobarbituric acid reactive substances (TBARS), catalase, UbcE2B expression, and total protein levels were evaluated using this aliquot.

#### 3.7.1. Estimation of Amyloid β1-42 Oligomer-Induced Changes of Acetylcholinesterase Activity

Tissue AChE levels were estimated using the method of Ellman (43). Briefly, 250 μL of 0.001 M Ellman’s reagent [5,5′-dithiobis-(2-nitrobenzoic acid); DTNB] was combined with 500 μL tissue aliquot. After adding 100 μL of 1 mM acetylthiocholine iodide, test tubes were incubated for 20 minutes at room temperature. Thiocholine reacts with DTNB to form a yellow chromogen, measured at 420 nm using a spectrophotometer (DU 640B Spectrophotometer, Beckman Coulter Inc., Brea, CA, USA). Acetylcholinesterase activity was calculated using the formula:


$$R\;=\frac{\delta \;O.D.\;\times \;Volume\;of\;the\;assay\;(3\;mL)}{\varepsilon \;\times \;mg\;of\;protein}$$

Where R is the rate of enzyme activity (n moles/min/mg protein), ε is the extinction coefficient (13,600/M/cm), and δ O.D. is the change in absorbance per minute.

#### 3.7.2. Estimation of Amyloid β1-42 Oligomer-Induced Changes of Thiobarbituric Acid Reactive Substances

The tissue TBARS levels were estimated as described by Ohkawa et al. (44). Briefly, 0.2 mL of an aliquot was combined with 1.5 mL of 30% acetic acid, 0.2 mL of sodium dodecyl sulfate (8.1%), and 1.5 mL of thiobarbituric acid (TBA: 0.8%). Distilled water was used to bring the total volume to 4 milliliters. Test tubes were then incubated at 90 °C for one hour. Additionally, 1 mL of distilled water was added, and the mixture was centrifuged for 10 minutes at 1968 G force. A spectrophotometer (DU 640B Spectrophotometer, Beckman Coulter Inc., Brea, CA, USA) set to 532 nanometers was used to quantify the variations in the pink-colored chromogen. About 0–10 nanomoles (nmol) of 1,1,3,3-tetramethoxypropane (TMP; also called malondialdehyde or TBARS test standards) per milliliter were used to create the reference standard plot. The outcome was expressed as MDA nmol per milligram of tissue protein.

#### 3.7.3. Estimation of Amyloid β1-42 Oligomer-Induced Changes of Reduced Glutathione

The tissue GSH levels were estimated as described by Beutler et al. (45). Briefly, 0.5 mL of an aliquot was combined with 2 mL of 0.3 M disodium hydrogen phosphate. Then, 0.25 mL of freshly prepared DTNB (0.001 M) was added. A spectrophotometer (DU 640B Spectrophotometer, Beckman Coulter Inc., Brea, CA, USA) set to measure absorbance at 412 nanometers was used to quantify the variations in the yellow-colored chromogen. About 10 - 100 µmol of GSH per milliliter was used to create the reference standard plot. The outcome was expressed as GSH µmol per milligram of tissue protein.

#### 3.7.4. Estimation of Amyloid β1-42 Oligomer-Induced Changes of Potassium-Chloride Co-Transporter 2 Expression

Tissue KCC2 levels were estimated using commercial enzyme-linked immunosorbent assay (ELISA) kits (MBS093618; MyBioSource, Selangor, Malaysia). Briefly, 50 μL tissue aliquot was added to a microplate, followed by 100 μL HRP-conjugate reagent. Plates were incubated for 60 minutes at 37°C, washed four times, then 50 μL chromogen solutions A and B were added. Plates were protected from light and incubated for 15 minutes at 37°C, followed by 50 μL stop solution. Absorbance was measured at 450 nm using a microplate reader (Bio-Tek Microplate Instruments, Penang, Malaysia). A standard curve was prepared using 0.625 - 20 ng/mL KCC2. Results were expressed as ng KCC2/mg tissue protein.

#### 3.7.5. Estimation of Amyloid β1-42 Oligomer-Induced Changes of Tissue Total Proteins

The tissue total proteins were estimated using the method described by Lowry et al. (46). Briefly, 5 mL of Lowry’s reagent, 1 mL of phosphate buffer, and roughly 0.15 mL of an aliquot were combined in test tubes and incubated for 15 minutes at room temperature. Then, 0.5 mL of Folin-Ciocalteu reagent was added and quickly vortexed, followed by 30 minutes of incubation at room temperature. A spectrophotometer (DU 640B Spectrophotometer, Beckman Coulter Inc., Brea, CA, USA) set to measure absorbance at 750 nanometers was used to measure the variations in the purple-colored chromogen. A standard curve was prepared using 0.2–2.4 mL bovine serum albumin. Results were expressed as mg total protein per g tissue.

### 3.8. Evaluation of Amyloid β1-42 Oligomer-Induced Histopathological Changes

Histopathological changes in the mouse hippocampal CA3 region were assessed using eosin-hematoxylin techniques as described by Dhamodharan et al. (47) with modification by Min Kaung Wint et al. (48). Tissue was fixed in 10% formalin and cut into coronal sections at 4 μm thickness. Changes were observed and images captured using an Olympus microscopic camera EP50 (Olympus Corporation, Tokyo, Japan). Microscopy was conducted at 400× magnification with a 35 μm scale bar.

### 3.9. Statistical Analysis

Standard deviations (SD) of each dataset were displayed. GraphPad Prism software version 5.0 (Dotmatics, R&D scientific software company, San Diego, CA, USA) was used for statistical analysis of MWM, WYMA, and NORT data using two-way analysis of variance (ANOVA) and Bonferroni post-hoc test. The Shapiro-Wilk test was used to assess data normality. One-way ANOVA and Tukey’s multiple range tests were used for AChE activity, TBARS, catalase, UbcE2B activity, and total protein levels. A probability value of less than 0.05 (P < 0.05) was considered statistically significant.

## 4. Results

### 4.1. Effect of Daphnetin on Amyloid β1-42 Oligomer-Induced Changes in Morris Water Maze Test Response

**Figure 1.**
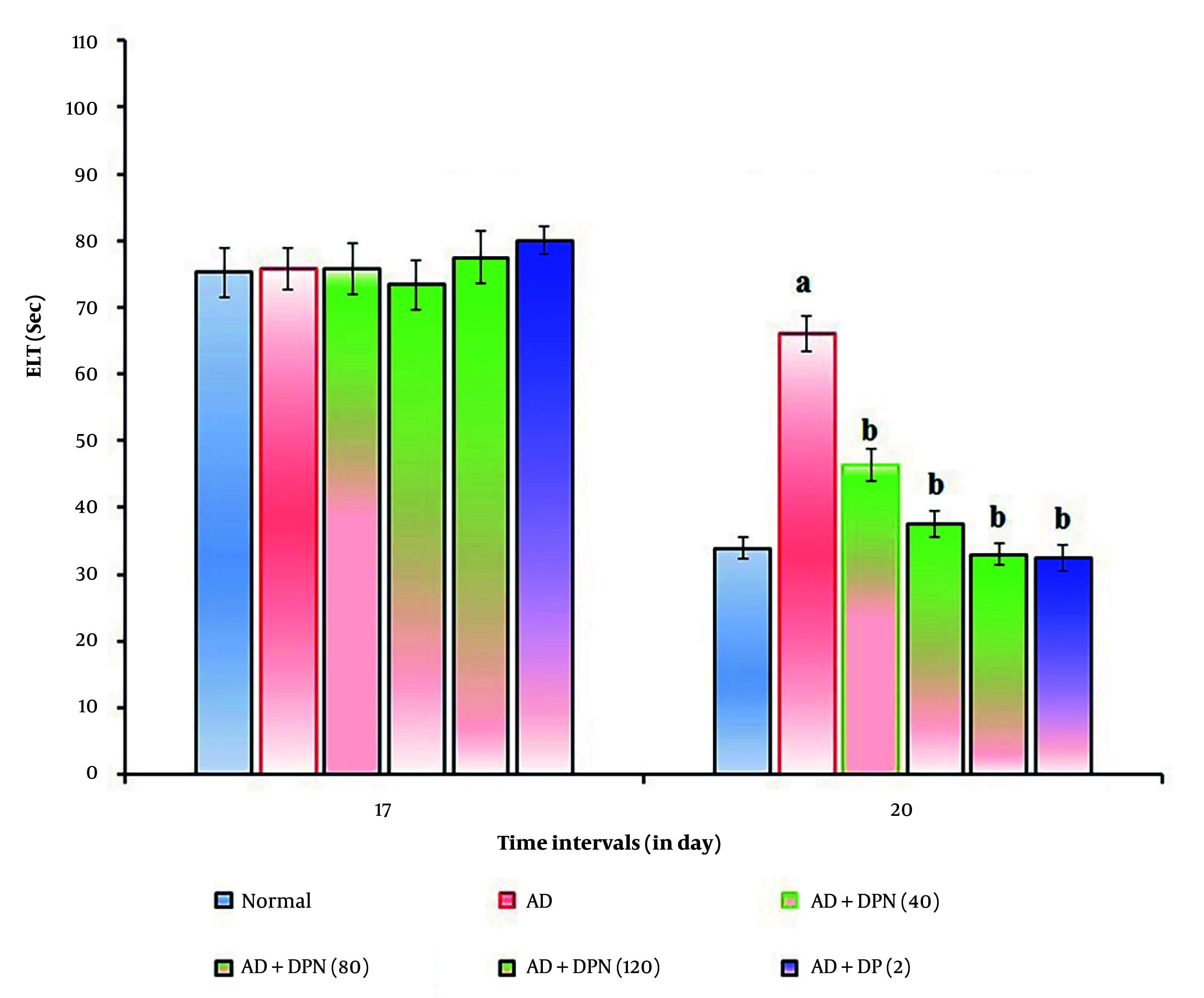
Effect of DPN on Aβ-induced changes of ELT values in the MWM test. Digits in parentheses indicate the dose in mg/kg. Data are expressed as the mean ± SD, n = 8 mice per group. a, P < 0.05 versus the normal group; b, P < 0.05 versus the AD group. Abbreviations: AD, Alzheimer's disease; DP, donepezil; DPN, daphnetin; ELT, escape latency time; and Sec, seconds.

In mice given Aβ (4 μg; i.c.v.), the MWM test revealed a significant (P < 0.05) increase in ELT and a decrease in TSTQ. Compared to the normal group, this suggests that Aβ may result in spatial memory impairments akin to AD due to downregulation of KCC2 and expression of neurotoxic (Aβ and tau) proteins. Oral administration of DPN (40, 80, and 120 mg/kg; p.o.; for 21 days) attenuated Aβ-induced AD in a dose-dependent manner (P < 0.05). The DP (2 mg/kg; p.o.; for 21 days) treatment group experienced a similar effect. Attenuation of DPN in the AD-induced rise of ELT and reduction of TSTQ values indicates that DPN possesses ameliorative potential against AD via regulation of cholinergic neurochemical alteration. The effect of DPN on Aβ-induced changes in ELT and TSTQ values in the MWM test are illustrated in Figures 1. and 2. 

**Figure 2.**
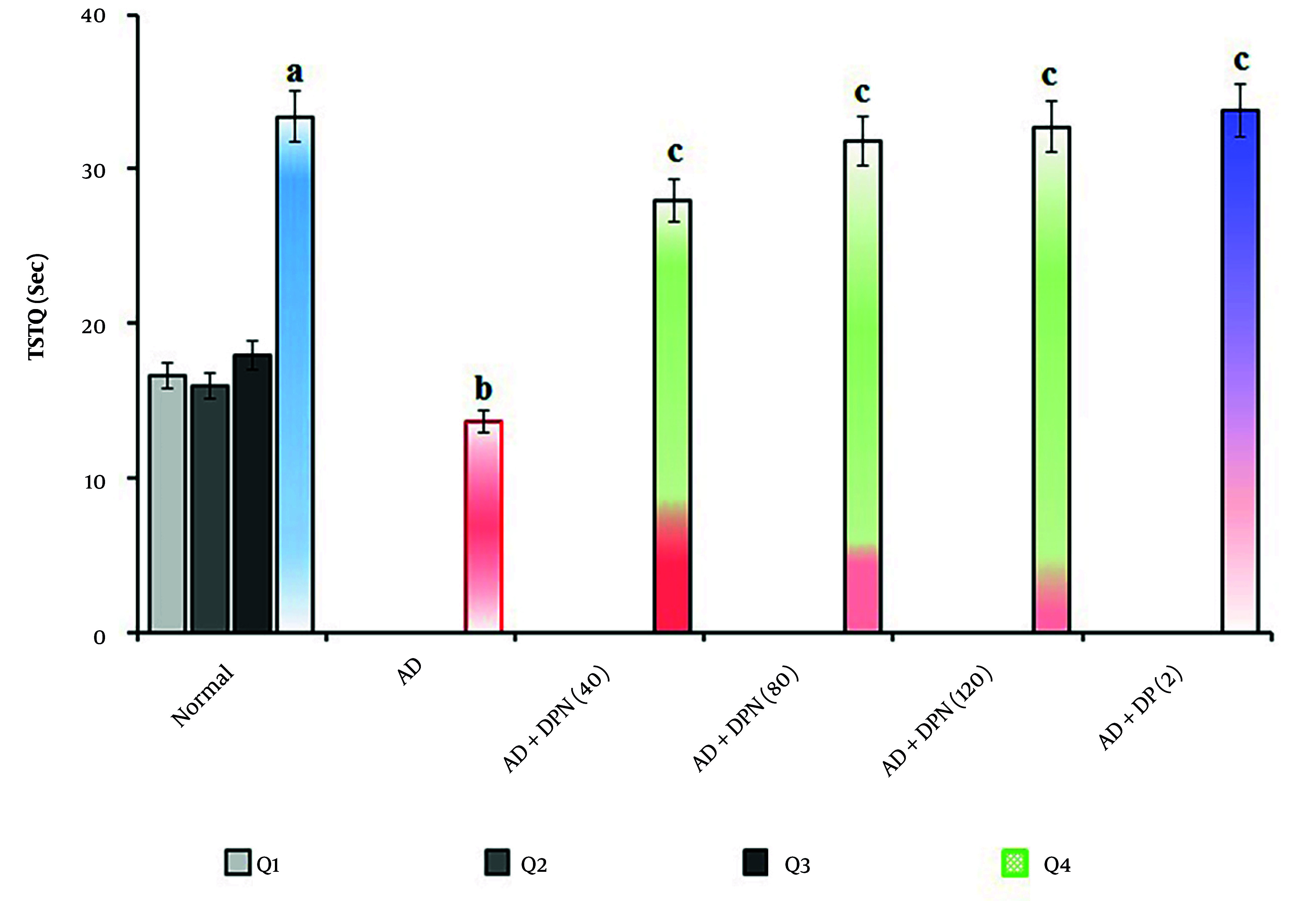
Effect of DPN on Aβ-induced changes of TSTQ values in the MWM test. Digits in parentheses indicate the dose in mg/kg. Data are expressed as the mean ± SD, n = 8 mice per group. a, P < 0.05 versus the Q1 of normal group; b, P < 0.05 versus the Q4 of normal group. c, P < 0.05 versus the Q4 of AD group. Abbreviations: AD, Alzheimer's disease; DP, donepezil; DPN, daphnetin; Sec, seconds; TSTQ, time spent in the target quadrant.

### 4.2. Effect of Daphnetin in Amyloid β1-42 Oligomer-Induced Changes of Water Y-Maze Alternation Test Response

**Figure 3.**
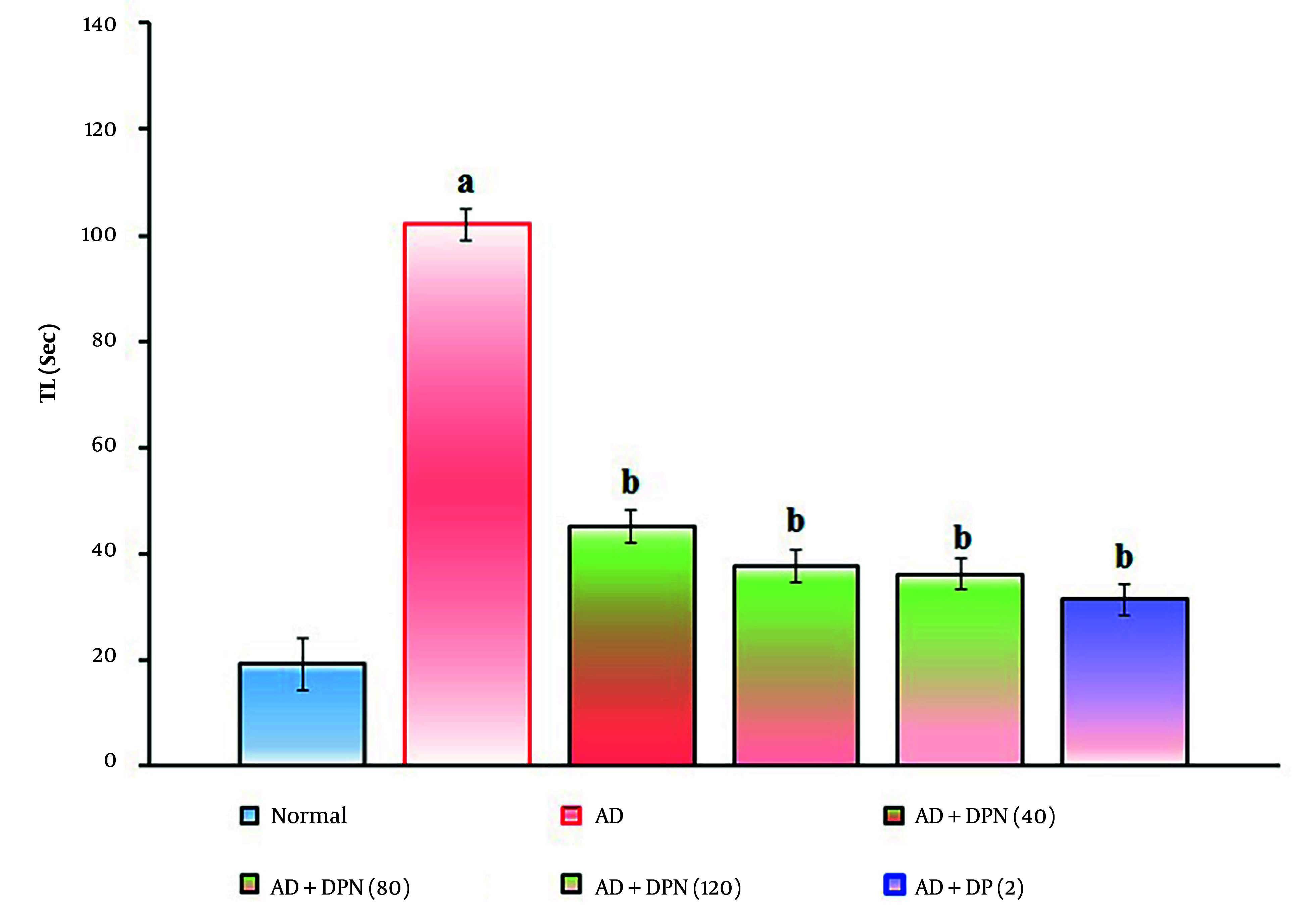
Effect of DPN on Aβ-induced changes of short-term memory functions. The numbers in parentheses represent a dose of mg/kg. The results are presented as the mean SD, with n = 8 mice per group. a, P < 0.05 versus the normal group; b, P < 0.05 versus the AD group. Abbreviations: AD, Alzheimer's disease; DP, donepezil; DPN, daphnetin; Sec, seconds; and TL, transfer latency.

Administration of Aβ (4 μg; i.c.v.) in mice showed a significant (P < 0.05) rise in TL in the WYMA test. Compared to the normal group, this suggests that Aβ may result in short-term memory loss akin to AD. Oral administration of DPN (40, 80, and 120 mg/kg; p.o.; for 21 days) attenuated Aβ-induced AD in a dose-dependent manner (P < 0.05). This effect was similar to the DP treatment group (2 mg/kg; p.o.; for 21 days). The attenuation of DPN in the AD-induced rise of TL values indicates that DPN possesses ameliorative potential against AD via regulation of cholinergic neurochemical alteration. The effect of DPN on Aβ-induced changes of TL values in the WYMA test is illustrated in Figure 3. 

### 4.3. Effect of Daphnetin in Amyloid β1-42 Oligomer-Induced Changes of Novel Object Recognition Test Response

**Figure 4.**
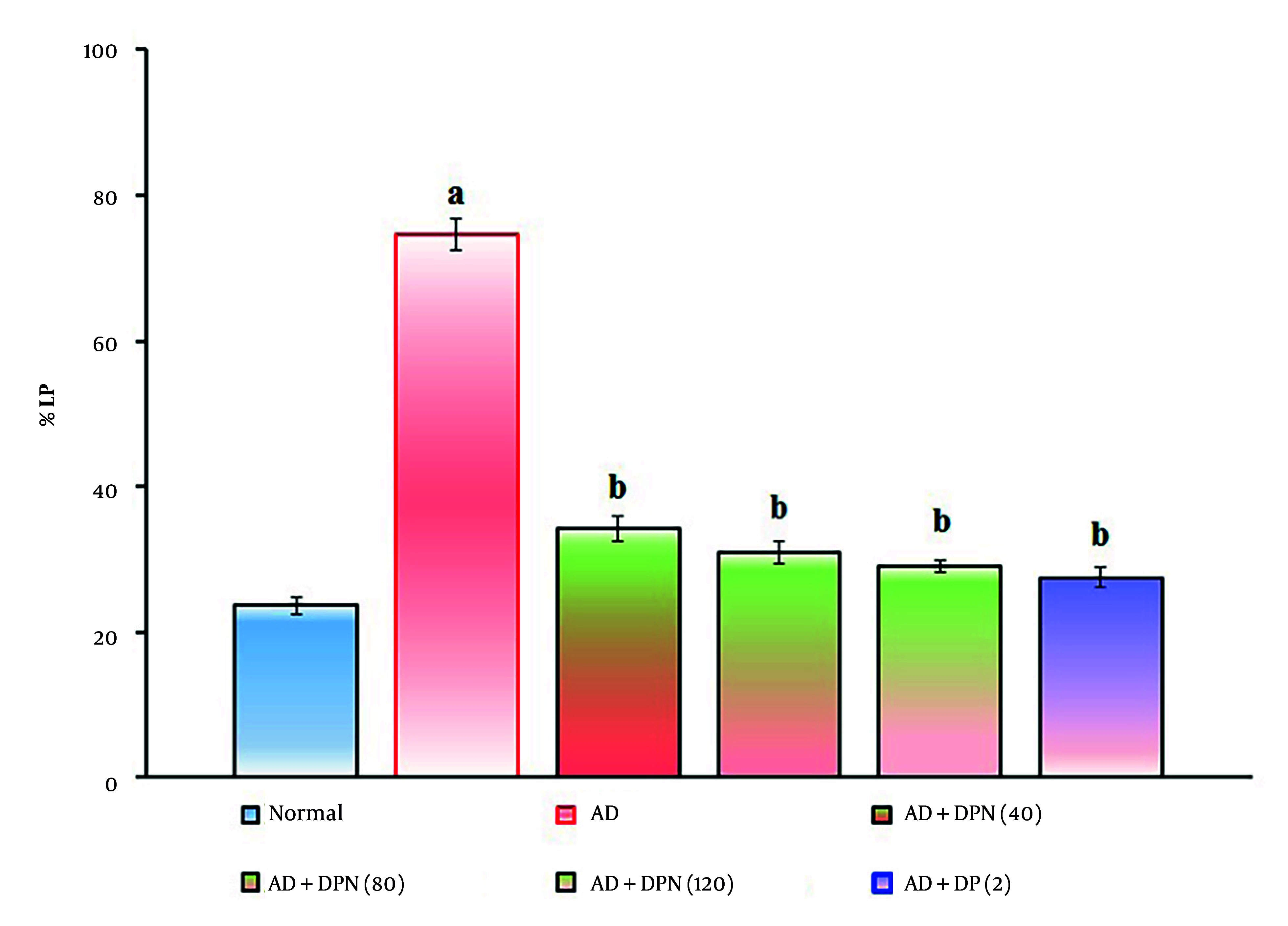
Effect of DPN on Aβ-induced changes of non-spatial learning functions. The numbers in parentheses represent a dose of mg/kg. The results are presented as the mean SD, with n = 8 mice per group. a, P < 0.05 versus the normal group; b, P < 0.05 versus the AD group. Abbreviations: AD, Alzheimer's disease; DP, donepezil; DPN, daphnetin; and %LP, percentage location preference.

Administration of Aβ (4 μg; i.c.v.) in mice showed a significant (P < 0.05) decrease in %LP and increase in %RI in the NORT device. Compared to the normal group, this suggests that Aβ may impair non-spatial learning function, similar to AD. Oral administration of DPN (40, 80, and 120 mg/kg; p.o.; for 21 days) attenuated Aβ-induced AD in a dose-dependent manner (P < 0.05). This effect was similar to the DP treatment group (2 mg/kg; p.o.; for 21 days). The attenuation of DPN in the AD-induced decrease in %LP and rise in %RI indicates that DPN possesses ameliorative potential against AD via regulation of cholinergic neurochemical alteration. The effect of DPN on Aβ-induced changes in %LP and %RI values in the NORT test is illustrated in Figures 4. and 5. 

**Figure 5.**
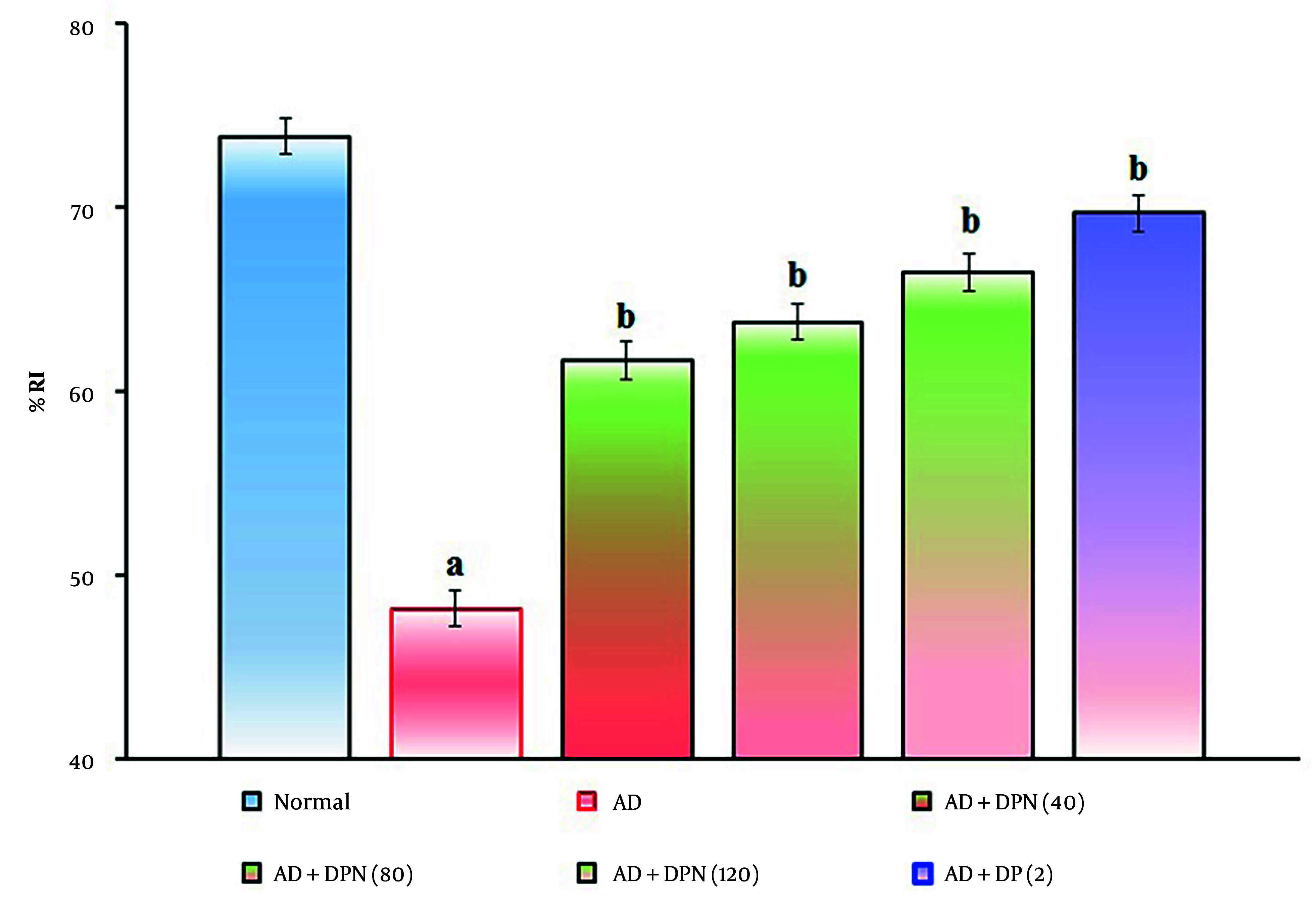
Effect of DPN on Aβ-induced changes of non-spatial learning functions. The numbers in parentheses represent a dose of mg/kg. The results are presented as the mean SD, with n = 8 mice per group. a, P < 0.05 versus the normal group; b, P < 0.05 versus the AD group. Abbreviations: AD, Alzheimer's disease; DP, donepezil; DPN, daphnetin; and %RI, percentage recognition index.

### 4.4. Effect of Daphnetin on Amyloid β1-42 Oligomer-Induced Tissue Biomarker Changes

In mice given Aβ (4 μg; i.c.v.), AChE activity and TBARS increased significantly (P < 0.05), while GSH and KCC2 levels decreased compared to the normal group. Oral administration of DPN (40, 80, and 120 mg/kg; p.o.; for 21 days) attenuated Aβ-induced AD in a dose-dependent manner (P < 0.05). This effect was similar to the DP treatment group (2 mg/kg; p.o.; for 21 days). The reduction of DPN in AD-induced alterations of these biomarkers suggests that DPN may prevent AD by controlling neuronal acetylcholine hydrolysis, neuronal membrane lipid peroxidation, oxidative stress, and neuronal DNA damage. Table 2 lists the impact of DPN on these tissue biomarker alterations caused by Aβ.

**Table 2.** Effect of Daphnetin on Aβ-Induced Tissue Biomarker Changes (n = 6) ^a,^ ^b^

Groups	AChE (μmol/mg Protein)	TBARS (nmol/mg Protein)	GSH (µmol/mg Protein)	KCC2 (ng/mg Protein)
**Normal**	19.7 ± 1.2	1.16 ± 0.07	8.61 ± 0.08	11.36 ± 0.12
**AD**	51.3 ± 0.9 ^b^	5.21 ± 0.12 ^b^	1.04 ± 0.14 ^b^	2.97 ± 0.09 ^b^
**AD+DPN (n = 40) (mg/kg)**	32.6 ± 1.1 ^c^	2.54 ± 0.17 ^c^	4.62 ± 0.16 ^c^	7.73 ± 0.14 ^c^
**AD+DPN (n = 80) (mg/kg)**	26.2 ± 1.4 ^c^	2.17 ± 0.09 ^c^	5.39 ± 0.08 ^c^	8.16 ± 0.05 ^c^
**AD+DPN (n = 120) (mg/kg)**	23.1 ± 0.6 ^c^	1.73 ± 0.06 ^c^	6.94 ± 0.14 ^c^	9.83 ± 0.08 ^c^
**AD+DP (n = 2) (mg/kg)**	20.3 ± 1.3 ^c^	1.52 ± 0.13 ^c^	7.69 ± 0.12 ^c^	10.19 ± 0.11 ^c^

Abbreviations: AChE, acetylcholinesterase; AD, Alzheimer’s disease; DP, donepezil; DPN, daphnetin; TBARS, thiobarbituric acid reactive substances; GSH, reduced glutathione; KCC2 , K⁺-Cl⁻ co-transporter 2.

^a^ Values are expressed as mean ± SD.

^b^ P < 0.05 versus normal group.

^c^ P < 0.05 versus AD group.

### 4.5. Effect of Daphnetin in Amyloid β1-42 Oligomer-Induced Histopathological Changes

**Figure 6.**
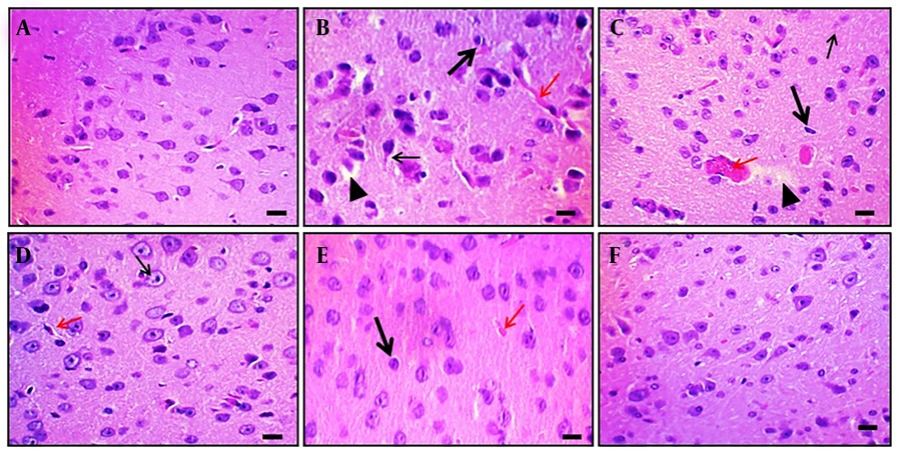
Effect of DPN on Aβ-induced histopathological changes of brain tissue in mice. Two mice were utilized in each group to evaluate the histological alterations in the brain tissue. Hematoxylin and eosin staining techniques were applied to tissue sections.A-F, histological changes of hippocampal tissue of naïve control, Aβ (4 μg; i.c.v.), DPN (40 mg/kg; for 21 days), DPN (80 mg/kg; for 21 days), DPN (120 mg/kg; for 21 days), and DP (2 mg/kg: p.o.; for 21 days) administered groups respectively. A, normal neuronal tissue structure. B, the Aβ-associated neuronal damage i.e., loss of neurons and neurofibrillary degeneration (thin arrow), nuclear pyknosis (thick arrow), vascular edema (red arrow), and vacuolation (arrowhead). C-E, the ameliorative action of DPN in a dosedependent manner against the Aβ neurotoxin. The possible neuroprotective effects of DP are depicted in F as being comparable to those of normal tissue. Microscopic analyses were conducted with a scale bar of 35 μm and a 400× magnification.

Aβ (4 μg; i.c.v.) administration caused microscopical changes in rat brain tissue, such as neuronal loss, neurofibrillary degeneration, nuclear pyknosis, vascular edema, and vacuolation. In contrast, naive control animals showed no changes. Oral DPN treatment (40, 80, and 120 mg/kg; p.o.; for 21 days) reduced Aβ-induced histological alterations. The outcomes were comparable to those of the DP treatment group (2 mg/kg; p.o.; for 21 days). This indicates that DPN possesses neuroprotective action against Aβ-induced neuronal damage and dysfunction. Observations were made at 400× magnification (scale bar: Thirty-five µm). Figure 6 shows the amelioration of DPN against Aβ-induced histopathological changes.

## 5. Discussion

Intracerebroventricular administration of Aβ (4 μg/4 μL) in mice caused significant (P < 0.05) changes in learning and memory patterns, i.e., increased ELT and reduced TSTQ in the MWM test. Aβ administration also resulted in increased TL in the WYMA test and decreased %LP and increased %RI in the NORT test. Biomarkers were also aggravated, with increased AChE activity and TBARS, and decreased GSH and KCC2 levels. These findings indicate that major changes in AD-associated neurovascular complications are mediated by oxidative stress, imbalance of cholinergic neurotransmission, and restoration of neuronal KCC2 channel, as evidenced by previous studies (16, 47, 49). Oral administration of DPN (40, 80, and 120 mg/kg; p.o.; for 21 days) attenuated these Aβ-associated AD pathologies in a dose-dependent manner, similar to the DP treatment group (2 mg/kg; p.o.; for 21 days). Furthermore, Aβ oligomer caused neuronal loss, neurodegeneration, nuclear pyknosis, vascular edema, and vacuolation, whereas DPN attenuated these histopathological changes due to its regulatory effects on acetylcholine hydrolysis, free radical formation, lipid peroxidation, reduced glutathione, and neuronal KCC2 homeostasis.

Our results indicate that restoration of the neuronal KCC2 channel is essential for preventing the generation of AD prion proteins, i.e., Aβ and tau, and neurofibrillary tangle formation (50). Neuronal KCC2 channels also regulate inhibitory GABAergic neurotransmission and the balance of excitatory neurotransmitters, which are implicated in the pathogenesis of various neurodegenerative disorders, including AD (51-53). The KCC2 channel also contributes to transcriptional and post-translational modifications of cellular survival factors such as neurotrophic factors, growth factors, apoptotic factors, cytokines, chemokines, and neurotransmitters, as well as avoidance of cell death factors like oxidative stress, excitotoxicity, and inflammatory proteins (54-56). Insulin also ameliorates aging-associated neurodegeneration in experimental animals via reduction of glial fibrillary acidic protein expression and inhibition of apoptosis (57). Potassium-chloride co-transporter 2 channels play multifaceted roles in neuron development by regulating neuronal survival and reciprocal regulation of synaptic activity (58, 59). In pathological conditions, especially neuronal stress, functional downregulation of neuronal function occurs via phosphorylation of the KCC2 channel (60). Conversely, neuronal oxidative stress and lipid peroxidation are associated with altered KCC2 channels and GABAergic signaling pathways (61). Similar results were observed in the present study, where Aβ peptides increased TBARS and reduced GSH levels.

Potassium-chloride co-transporter 2 and AChE proteins have distinct roles: Potassium-chloride co-transporter 2 inhibits neuronal KCC2 channel functions, whereas AChE breaks down acetylcholine neurotransmitter (62). Regulation of both KCC2 and AChE is essential for memory formation and consolidation via maintaining synaptic plasticity and enhancing cholinergic neurotransmission, respectively (63). Potassium-chloride co-transporter 2 supports memory function, while AChE facilitates memory processes in mice (28). The current data revealed that Aβ increases ELT and reduces TSTQ in the MWM test, raises TL in the WYMA test, and reduces %LP and increases %RI in the NORT test. The natural coumarin derivative DPN demonstrated nootropic action against Aβ-induced cognitive dysfunction in mice, consistent with previous reports on DPN’s cognitive improvement in diabetic animals (30, 31) and neuroprotective actions with potassium channel modulatory effects (29). Natural pomegranate seed oil also attenuates Aβ42-induced AD in rats by restoring neuronal density proteins (64). Donepezil, in addition to its anti-cholinergic action, is known to possess neuroprotective effects via regulation of neuronal KCNQ2 and KCNQ3 channels (27).

Lack of neuronal KCC2 function enhances neuronal hyperexcitability and neurodegeneration in neurological disorders, whereas restoration of KCC2-mediated chloride extrusion helps maintain low intracellular chloride, enabling inhibitory GABAergic neurotransmission and neuroprotection (65). Neuronal KCC2 function modulates axonal degeneration, demyelination, and neuronal regeneration (66). In this study, Aβ oligomer administration altered neuronal function, biomarkers, and histology, while DPN treatment attenuated these effects. Literature reports confirm that DPN possesses neuroprotective action and maintains blood-brain barrier integrity against cerebral ischemic insult in mice (30). The current study also demonstrated that DPN attenuates Aβ oligomer-associated neurotoxicity and neuronal dysfunction.

### 5.1. Conclusions

This study revealed that oral administration of DPN ameliorates Aβ oligomer-associated cognitive dysfunction in mouse models of AD. Furthermore, DPN provides neuroprotection via regulation of cellular oxidative stress, inflammation, and restoration of KCC2 channel functions. Hence, DPN may be useful for the treatment of AD. However, further evaluation in different AD conditions and animal species is warranted to confirm its therapeutic potency and targeted actions.

## Data Availability

The dataset used and analyzed in the current study is available upon request.
